# Oral administration of *Pantoea agglomerans*-derived lipopolysaccharide prevents development of atherosclerosis in high-fat diet-fed apoE-deficient mice via ameliorating hyperlipidemia, pro-inflammatory mediators and oxidative responses

**DOI:** 10.1371/journal.pone.0195008

**Published:** 2018-03-27

**Authors:** Yutaro Kobayashi, Hiroyuki Inagawa, Chie Kohchi, Kimiko Kazumura, Hiroshi Tsuchiya, Toshiyuki Miwa, Katsuichiro Okazaki, Gen-Ichiro Soma

**Affiliations:** 1 Departments of Integrated and Holistic Immunology, Faculty of Medicine, Kagawa University, Kagawa, Japan; 2 Control of Innate Immunity, Technology Research Association, Kagawa, Japan; 3 Research Institute for Healthy Living, Niigata University of Pharmacy and Applied Life Sciences, Niigata, Japan; 4 Macrophi Inc., Kagawa, Japan; 5 Central Research Laboratory, Hamamatsu Photonics K.K., Shizuoka, Japan; 6 Department of Applied Biological Science, Faculty of Agriculture, Kagawa University, Kagawa, Japan; The University of Tokyo, JAPAN

## Abstract

*Pantoea agglomerans* (*P*. *agglomerans*) is a Gram-negative bacterium that grows symbiotically with various edible plants, and the oral or sublingual administration of lipopolysaccharide derived from *P*. *agglomerans* (LPSp) have been suggested to contribute to prevention of immune-related diseases. Our previous study indicated that orally administered LPSp was shown to exhibit an LDL-lowering effect in hyperlipidemic volunteers; however, a preventive effect of LPSp on atherosclerosis is unclear. The present study attempted to evaluate the anti-atherosclerotic effect by LPSp in a mouse model of high-fat diet (HFD)-induced atherosclerosis. For 16 weeks, apoE-deficient mice were fed an HFD and received drinking water containing LPSp (0.3 or 1 mg/kg body weight/day). The results showed that the orally administered LPSp decreased body weight. A significant reduction in atherosclerotic plaque deposition was observed even with the lower dose of LPSp. The biochemical analyses showed that LPSp markedly improved glucose tolerance and reduced plasma LDL and oxidized LDL levels. In addition, LPSp significantly reduced the production of pro-inflammatory mediators including MCP-1 (in the plasma), TNF-α and IL-6 (in the colon), and decreased the oxidative burst activities in the peripheral blood sample. Taken together, these results suggest the possibility that oral administration of LPSp can effectively ameliorate HFD-induced hyperlipidemia and inflammatory/oxidative responses to prevent atherosclerosis and related metabolic disorders.

## Introduction

Lipopolysaccharide (LPS), which is the major component of the outer membrane of Gram-negative bacteria, is well known as a strong immunostimulant in both humans and animals. However, recent studies have demonstrated some beneficial effects on the prevention of various disorders. Many epidemiological studies have confirmed that environmental exposure to LPS in early life, such as with farm living, decreased the risk of allergic sensitization, asthma and hay fever in childhood [1]. Also, pretreatment with low-dose LPS *via* intravenous injection was found to enhance resistance to *Staphylococcus aureus* infection in a mouse model with possible mechanisms of activation (priming) of macrophages *via* epigenetic changes by LPS-mediated toll-like receptor (TLR) 4 signaling pathways [2].

Our group has been investigating the physiological effects of macrophage (or immune cell)-activating compounds obtained from edible plants and associated by-products [3–5]. We previously found that a water extract of wheat flour exhibited *in vivo* priming activity in mice and its active compound was determined as LPS originating from *Pantoea agglomerans* (*P*. *agglomerans*) attached to the wheat [6]. *P*. *agglomerans* is a Gram-negative bacterium that grows symbiotically with various edible plants, such as wheat and rice [7], and several strains have been used as biological control agents for prevention of bacterial diseases on pome fruits [8]. *In vivo* studies have reported that oral or sublingual administration of LPS purified from *P*. *agglomerans* (LPSp) is associated with several important health benefits, including prevention against gastric ulcer [9], atopic dermatitis [10] and diabetes [11] and improvement mucosal vaccine potency against influenza virus [12]. The safety of LPSp when administered *via* the oral or transdermal route was confirmed by common *in vivo* safety tests and skin patch tests [13].

Atherosclerosis remains one of the leading causes of morbidity and mortality worldwide and hyperlipidemia is known to be a major risk factor for the development of atherosclerosis [14]. In the early stage of atherosclerosis, production of oxidized LDL (ox-LDL) and formation of atherosclerotic plaque is mainly caused by activated vessel immune cells with excessive inflammatory responses provoked by pathogen recognition receptors, including TLRs; however, findings of TLR-mediated atheroprotective effects have also emerged [15]. For example, a heat shock protein, which acts as TLR4 agonist, was found to show preventive effects against hyperlipidemia and atherosclerosis when administered orally to apoE-deficient mice [16]. In our previous clinical study, oral administration of the LPSp-containing tea resulted in a significant reduction in levels of fasting plasma glucose and low-density lipoprotein (LDL) cholesterol in hyperlipidemic volunteers with no adverse effects [17]. In addition, orally administered LPSp was shown to be effective in lowering a serum LDL level in a rabbit model of hyperlipidemia (Watanabe hereditary hyperlipidemic rabbits) [18], however, no report has yet to describe the anti-atherosclerotic effect by orally administered LPSp.

Therefore, the aim of the present study was to determine whether oral administration of LPSp prevents the development of atherosclerosis in a high-fat diet (HFD)-induced model of atherosclerosis in apoE-deficient mice. Orally administered LPSp for 16 weeks significantly reduced the atherosclerotic plaque deposition in the thoracic aorta. In addition, LPSp markedly improved glucose tolerance and reduced plasma LDL, oxidized LDL levels, pro-inflammatory mediators, and the oxidative burst activities in the peripheral blood sample. Our findings firstly demonstrated that LPSp can effectively ameliorate hyperlipidemia and inflammatory/oxidative responses to prevent atherosclerosis progression when administered orally to HFD-fed apoE-deficient mice.

## Methods

### Animal experiments

Male apoE-deficient mice (BALB/c.KOR/StmSlc-*Apoe*^*shl*^), aged 10–12 weeks, were purchased from Japan SLC, Inc. (Shizuoka, Japan) and maintained in a temperature- and humidity-controlled room under a 12-h light/dark cycle with unrestricted access to food and water. A low-fat diet (LFD; 16.1 kJ/g, 4.3% w/w fat and 0.005% w/w cholesterol; D12450B) and HFD (21.9 kJ/g, 35% w/w fat and 0.03% w/w cholesterol; D12492) were obtained from Research Diets, Inc. (New Brunswick, NJ, USA). All mice were acclimated for 1 week while being fed a LFD and receiving tap water. Mice were assigned to three groups (*n* = 8 each) and fed a HFD for 16 weeks. Purified LPS derived from *P*. *agglomerans* (LPSp; Macrophi Inc., Kagawa, Japan) was dissolved in drinking water and applied at 0.3 or 1 mg/kg body weight (BW)/day. The dose of LPSp was determined to be the sufficient dose required to achieve preventive effects as estimated from previous *in vivo* studies (0.1–1 mg/kg BW/day) [10, 19]. The drinking water was changed weekly and the concentration of LPSp was adjusted according to the average BW and amount of water consumption. We confirmed that the LPSp in drinking water was not significantly degraded in a week (S1 Fig). The water sample containing LPSp (3 or 10 μg/ml, *n* = 3) was prepared and stored at 25°C. The concentration was approximately equivalent to doses of 0.3 or 1 mg/kg BW/day, respectively. An aliquot of water sample was collected at day 0, 3, 5 and 7. The concentration of LPSp in the sample was determined by the Limulus Amebocyte Lysate (LAL) assay reagents and PyroColor Diazo Reagents (Associates of Cape Cod Inc., East Falmouth, MA), according to the manufacturerer’s protocol. The BW, food intake and water intake were recorded weekly. At week 16, the mice were fasted overnight and subjected to an oral glucose tolerance test (OGTT) by oral glucose administration (gavage with 2 g of _D_-glucose/kg BW). Blood samples were collected from the tail vein and blood glucose levels were monitored using an Accu-Chek Aviva blood glucose meter with Accu-Chek Aviva test strips (Roche Diagnostics K.K., Quebec, Canada) at 0, 15, 30, 60 and 120 min after glucose loading. The area under the curve (AUC) was calculated using the trapezoid rule. At the end of week 16, fresh stool samples were collected and stored at −80°C for further analysis and the mice were anaesthetized under sevoflurane vapor after overnight food deprivation. Blood samples were collected *via* heart puncture with a syringe rinsed with heparin. The whole blood samples were used for further analysis within 6 h after collection. Plasma was collected after centrifugation (1,200 × *g* for 20 min at 4°C) and stored at −80°C. Liver and epididymal white adipose tissues were removed and weighed. The thoracic aorta was carefully removed and stored in freshly prepared fixation solution (4% paraformaldehyde in phosphate-buffered saline) at 4°C for 24 h. The animal experiment protocols were approved by the Animal Care and Use Committee of Kagawa University (approval no. 16621). This experiment was carried out according to the guidelines for animal experiments at the Faculty of Medicine, Kagawa University.

### Assessment of atherosclerotic lesions in the thoracic aorta

Quantification analysis of atherosclerotic lesions in the thoracic aorta was performed as described previously [20]. Briefly, the fixed aorta was stained with 0.2% w/v Oil red O solution for 60 min. This solution was freshly prepared by dissolving Oil Red O (Wako Pure Chemical Industries, Ltd., Osaka, Japan) in methanol and filtered through　a membrane (0.45 μm) before use. The stained aorta was opened longitudinally, pinned on a dissecting dish and the intimal surface was digitally photographed. The percentage of Oil red O-positive plaque coverage on the total thoracic area was quantified using Image J software [21].

### Biochemical measurements

Plasma insulin levels were determined with a mouse insulin ELISA kit (AKRIN-011S; Shibayagi, Gunma, Japan) according to the manufacturer’s instructions. Hemoglobin A1c (HbA1c) was measured with an HbA1c measurement kit (Sekisui Medical, Tokyo, Japan). Plasma triglyceride, LDL, high-density lipoprotein (HDL), total cholesterol, non-esterified cholesterol, aspartate aminotransferase (AST) and alanine aminotransferase (ALT) levels were determined with commercial kits (Wako Pure Chemical Industries). Plasma esterified cholesterol was calculated by subtracting non-esterified cholesterol from total cholesterol. Plasma levels of ox-LDL were determined with a mouse Ox-LDL ELISA kit (Kamiya Biomedical Company, Tukwila, WA, USA). Cytokines were measured using commercial mouse ELISA kits (Affymetrix, Santa Clara, CA, USA): tumor necrosis factor (TNF)-α (#88–7324), interleukin (IL)-6 (#88–7064) and monocyte chemoattractant protein (MCP)-1 (#88–7391). The colon tissue was homogenized in PBS containing 1% protease inhibitor cocktail (GE Healthcare UK Ltd., Buckinghamshire, England) for 3 min using a homogenizer. The supernatant was collected following centrifugation at 10,000 × *g* for 10 min at 4°C. The concentration of colonic cytokines is reported as picograms of cytokine relative to protein content (mg of protein). The protein concentration was determined by a bicinchoninic acid (BCA) assay (Thermo Fisher Scientific, Waltham, MA, USA). Plasma levels of high molecular weight (HMW) adiponectin were determined with a mouse/rat HMW adiponectin ELISA kit (Shibayagi). Lipid extraction from the liver tissues was performed with the butanol-methanol method as described previously [22] with some modifications. Briefly, individual snap-frozen liver samples were weighted and homogenized in chilled butanol/methanol (3:1 v/v) containing six zirconium oxide beads with a vortex mixer for 15 min. The homogenates were mixed with heptane/ethyl acetate (3:1 v/v) and acetic acid (1% v/v) and then centrifuged at 4,000 × *g* for 10 min at 4°C. The upper phase was collected and heptane/ethyl acetate (3:1 v/v) was added again to the lower phase, which was vortexed for 15 min. After centrifugation, the upper phase was collected and pooled with the previous collection. The lipid extracts were stored at −80°C until use. Hepatic triglyceride levels were measured with a triglyceride measurement kit (Wako Pure Chemical Industries). The total abundance of LPS in plasma was analyzed by a kinetic turbidimetric assay using the Limulus Amebocyte Lysate (LAL) assay kit and a Toxinometer® ET-6000 computer-operated kinetic incubating tube reader (Wako Pure Chemical Industries) with the following modifications: the plasma was diluted to 1:10 in pyrogen-free water and preheated to 70°C for 10 min prior to analyses. The standard LPS (Lot: EBJ0398) contained in the LAL kit was used here and the standard curve was plotted with log concentration of each standard against log reaction time according to the manufacturer’s instructions.

### Simultaneous measurement of superoxide anion (O_2_^●-^) production and myeloperoxidase (MPO) activity

O_2_^●-^ production and MPO activity after stimulation with phorbol 12-myristate 13-acetate (PMA; Wako Pure Chemical Industries) in the hemolyzed blood sample was simultaneously determined with a real-time chemiluminescence and fluorescence monitoring system (CFL-P2200; Hamamatsu Photonics K.K., Shizuoka, Japan), comprised of a high speed on/off system with LED excitation light and a chemiluminescence/fluorescence high sensitivity detector system, as previously described [23,24] with some modification. An aliquot of each whole blood sample (30 μl) was mixed with 500 μl of an ammonium chloride-based red blood cells lysis buffer with no fixing reagents (Tonbo Biosciences, San Diego, CA, USA) for 2 min at room temperature (RT). After centrifugation (200 × *g* for 5 min at RT), the cell pellet was suspended in Ringer–Hepes buffer (154 mM NaCl, 5.6 mM KCl and 10 mM Hepes, pH 7.4). The cell suspension was pre-incubated with 1 mM CaCl_2_, 0.5 μM MCLA (6-(4-methoxyphenyl)-2-methyl-3,7-dihydroimidazo[1,2-a]pyrazin-3-one hydrochloride; Tokyo Chemical Industry Co., Ltd., Tokyo, Japan) and 2 μM aminophenyl fluorescein (APF; Goryo Chemical, Inc., Hokkaido, Japan) for 2 min at 37°C. Here, MCLA was used as an O_2_^●-^ sensitive chemiluminescence reagent and APF was used as a hypochlorous acid (the oxidation product by MPO reaction)-sensitive fluorescence reagent. The value of chemiluminescence intensity was calculated as relative chemiluminescence units (RCU) and fluorescence intensity as relative fluorescence units (RFU) with the CFL-P2200 system. Subsequently, RCU and RFU were monitored every 0.5 s. After 60 s, PMA (1 μM) was injected into the sample by an auto injector. The sample was continuously stirred at 37°C during the measurements. The PMA-induced responses were calculated as the peak intensity minus the basal value.

### Analysis of phagocytic activity

The phagocytic activity in the blood sample was monitored using the CFL-P2200 system. Briefly, 10 μl of pHrodo Green *E*. *coli* BioParticle conjugate (1 mg/ml; Thermo Fisher Scientific) was added to an aliquot of each blood sample (60 μl). Then, half of the mixture was incubated for 2 h at 37°C, while the other half was stored for 2 h at 4°C as a negative control. After incubation, the sample was hemolyzed as described above and the cell pellet was suspended in the Ringer–Hepes buffer. The fluorescence intensity of the sample was measured using the CFL-P2200 system and phagocytic activity was calculated as the fluorescence intensity of a sample incubated at 37°C minus that of a sample incubated at 4°C.

### Flow cytometric analysis of neutrophils and monocytes in the blood samples

An aliquot of each whole blood sample (30 μl) was incubated for 15 min on ice with the following antibodies (BioLegend, San Diego, CA, USA): PE-labelled anti-mouse CD45 (clone 30-F11), PE/Cy7 anti-mouse/human CD11b (clone M1/70), APC anti-mouse Ly-6G (clone 1A8) and APC/Fire750 anti-mouse Ly-6C (clone HK1.4). Negative control staining was performed using appropriate isotype antibodies (BioLegend). The stained blood samples were lysed with a red blood cells lysis buffer (Tonbo Biosciences) and analyzed using a Gallios flow cytometer and Kaluza software (Beckman Coulter, Inc., Brea, CA, USA). Cells were defined with the following stains [25]: the gated CD45^+^CD11b^+^ cells as total white blood cells; CD45^+^CD11b^+^Ly-6G^+^ as neutrophils; CD45^+^CD11b^+^Ly-6G^-^Ly-6C^+^ as monocytes. The gating strategy for these neutrophils and monocytes was summarized in S2 Fig.

### Microbial community analysis

Microbial DNA was extracted from mouse stools using the QIAamp Fast DNA Stool Mini Kit (Qiagen, Hilden, Germany) according to the manufacturer’s protocol. The amplification, sequencing and analysis of the extracted DNA were performed by Bioengineering Lab (Kanagawa, Japan). Briefly, the 16S rRNA V3-V4 region was amplified using 16S amplicon PCR primers (341f: 5’- ACA CTC TTT CCC TAC ACG ACG CTC TTC CGA TCT CCT ACG GGN GGC WGC AG-3’ and 805r: 5’- GTG ACT GGA GTT CAG ACG TGT GCT CTT CCG ATC TGA CTA CHV GGG TAT CTA ATC C-3’). Sequencing of the purified 16S rRNA amplicons was carried out on an Illumina Miseq platform (Illumina, Inc., San Diego, CA, USA). Post processing sequencing data were analyzed with QIIME software (version 1.9.0.) [26]. Quality filtering was performed using the default parameters in QIIME. Sequences were grouped into operational taxonomic units at a 97% sequence similarity threshold using the UCLUST algorithm [27].

### Statistical analysis

Data are expressed as the mean ± standard error of the mean (SEM). All statistical analyses were performed using GraphPad Prism version 7.0 software (Graphpad Software, Inc., La Jolla, CA, USA). Statistical differences between curves were analyzed using two-way analysis of variance (ANOVA) followed by Tukey’s multiple-comparison test. Statistical differences between multiple groups were determined using one-way ANOVA followed by Tukey’s multiple-comparison test. Correlation coefficient values were calculated using Pearson's correlation coefficient. A probability (*p*) value of less than 0.05 was considered statistically significant.

## Results

### General observations

**Fig 1:**
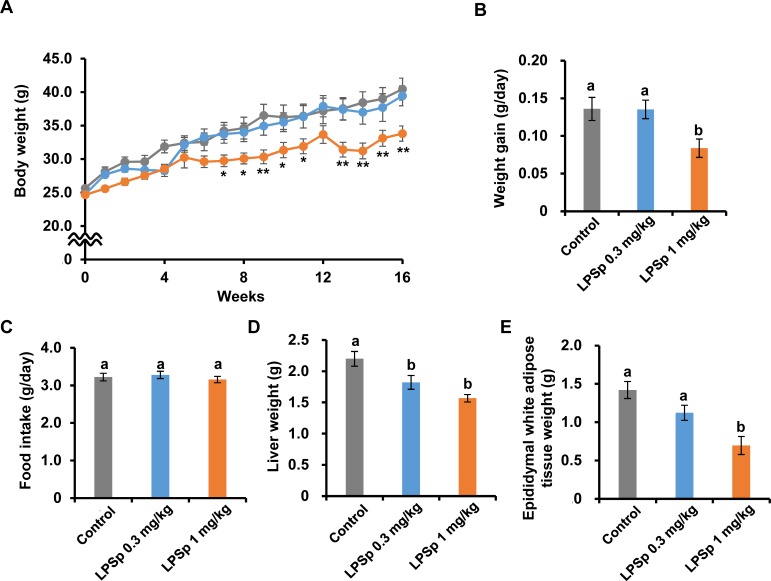
Effects of orally administered LPSp on BW changes, daily weight gain, food intake and liver and epididymal white adipose tissue weights. (A–C) Body weight (BW) and food intake were monitored weekly and daily weight gain was calculated over the experimental period. (D–E) After the experiment, the weight of liver and epididymal white adipose tissues were measured. Values are presented as the mean ± SEM, *n* = 8. A single asterisk (*) and double asterisks (**) indicate statistically significant differences (*p* < 0.05 and *p* < 0.01 respectively) as compared with the control group (two way ANOVA, post-hoc Tukey test). Unless indicated, no significance difference was observed between groups. Different letters indicate statistically significant difference between treatments (*p* < 0.05, one way ANOVA, post-hoc Tukey test).

To address the effect of LPSp on atherosclerosis, apoE-deficient mice were fed a HFD to induce development of atherosclerosis and received drinking water spiked with purified LPSp for 16 weeks. Mice received LPSp at a dose of 0.3 or 1 mg/kg BW/day based on BW and water intake. As shown in Fig 1A, the BW of all mice increased gradually over time. Oral administration of LPSp (1 mg/kg BW/day) remarkably alleviated the HFD-induced weight gain from week 7 to 16 (*p* < 0.05), except for week 12 (*p* = 0.12). Likewise, the daily weight gain was reduced in the LPSp (1 mg/kg BW/day)-treated group, as compared to the control group (*p* < 0.05) (Fig 1B). There were no significant differences among groups in food intake during the experiment (Fig 1C and S3 Fig) or initial BW (control: 25.6 ± 0.4 g; LPSp 0.3 mg/kg BW/day: 24.7 ± 0.3 g; LPSp 1 mg/kg BW/day: 24.7 ± 0.4 g, *n* = 8). As compared to the control group, the liver weight was lower in the LPSp-treated groups (LPSp 0.3 mg/kg BW/day: *p* < 0.05; LPSp 1 mg/kg BW/day: *p* < 0.01) (Fig 1D) and the epididymal white adipose tissue was much lower in the LPSp (1 mg/kg BW/day)-treated group (*p* < 0.05) (Fig 1E). During the experiment, no mouse died and there was no incidence of adverse phenomena, such as rectal bleeding, diarrhea or abnormal behaviors.

### Oral glucose tolerance test, fasting insulin and HbA1c

**Fig 2:**
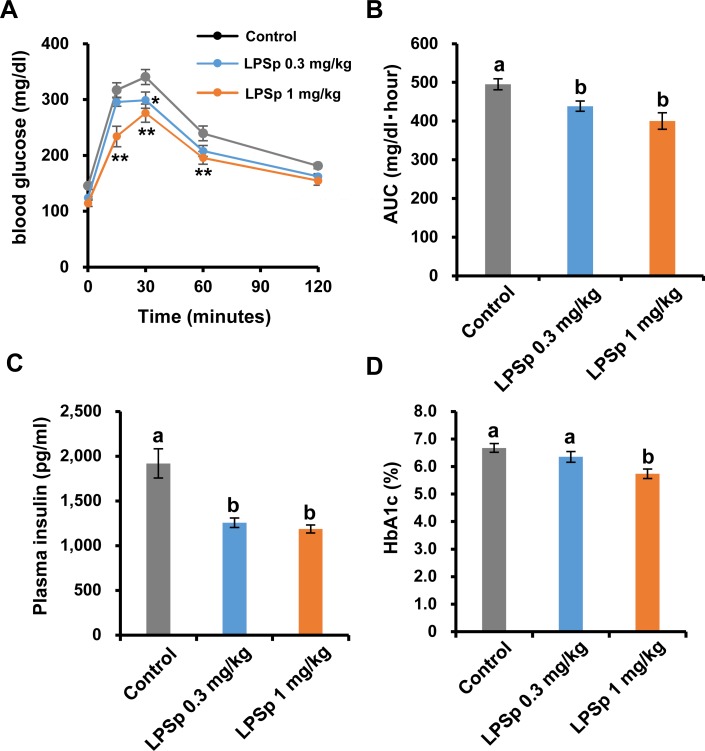
Effects of orally administered LPSp on OGTT response, plasma insulin and HbA1c. (A–B) In order to evaluate glucose tolerance, an oral glucose tolerance test (OGTT) test was performed at week 16. The glucose level in blood obtained from the tail vein was measured at 0, 15, 30, 60 and 120 min after glucose loading (2 g _D_-glucose/kg BW). The AUC was calculated using the trapezoid rule. (C–D) The fasting plasma insulin and HbA1c levels were measured using commercial kits after the experiment. Values are presented as the mean ± SEM, *n* = 8. A single asterisk (*) and double asterisks (**) indicate statistically significant differences (*p* < 0.05 and *p* < 0.01 respectively) as compared with the control group (two way ANOVA, post-hoc Tukey test). Unless indicated, no significance difference was observed between groups. Different letters indicate statistically significant difference between treatments (*p* < 0.05, one way ANOVA, post-hoc Tukey test).

Development of atherosclerosis is associated with worsened glucose tolerance, lipid and adipokine profiles, as well as inflammatory biomarkers [28]. To evaluate the effect of LPSp on glucose tolerance, an OGTT was performed at week 16. As shown in Fig 2A, oral administration of LPSp (1 mg/kg BW/day) induced a pronounced reduction in the blood glucose response to the glucose load at 15, 30 and 60 min as compared to the control group (*p* < 0.01). The lower dose of LPSp (0.3 mg/kg BW/day) induced a significant decrease in blood glucose at 30 min (*p* < 0.05). The AUC analysis demonstrated a significant reduction in blood glucose in both LPSp-treated group as compared to the control group (LPSp 0.3 mg/kg BW/day: *p* < 0.05; LPSp 1 mg/kg BW/day: *p* < 0.01) (Fig 2B). After the treatment, the fasting plasma insulin level was lower in both LPSp-treated groups as compared to the control group (*p* < 0.01) (Fig 2C) and HbA1c was also lower in the LPSp (1 mg/kg BW/day)-treated group (*p* < 0.01) (Fig 2D).

### Quantitative analysis of atherosclerotic lesion in the thoracic aorta

**Fig 3:**
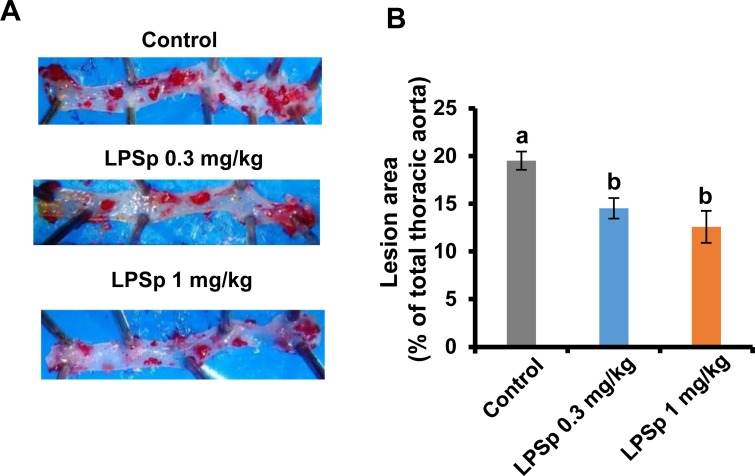
Effects of orally administered LPSp on atherosclerotic lesion area in the thoracic aorta. The paraformaldehyde-fixed thoracic aorta was stained with Oil Red O. (A) Representative photographs of the intimal surface of the stained thoracic aorta. (B) The percentage of the atherosclerotic lesion area was quantified by image analysis based on Oil red O-positive plaque coverage on the thoracic aorta. Values are presented as the mean ± SEM, *n* = 8. Different letters indicate statistically significant difference between treatments (*p* < 0.05, one way ANOVA, post-hoc Tukey test).

After 16 weeks of oral LPSp administration, quantitative analysis of the thoracic aorta showed that there were fewer Oil Red O-positive atherosclerotic lesions in the LPSp-treated group as compared to the control group (LPSp 0.3 mg/kg BW/day: *p* < 0.05; LPSp 1 mg/kg BW/day: *p* < 0.01) (Fig 3A and 3B). Between the two LPSp-treated groups, there was no significant dose-dependent reduction of the plaque area.

### Biochemical parameters

**Fig 4:**
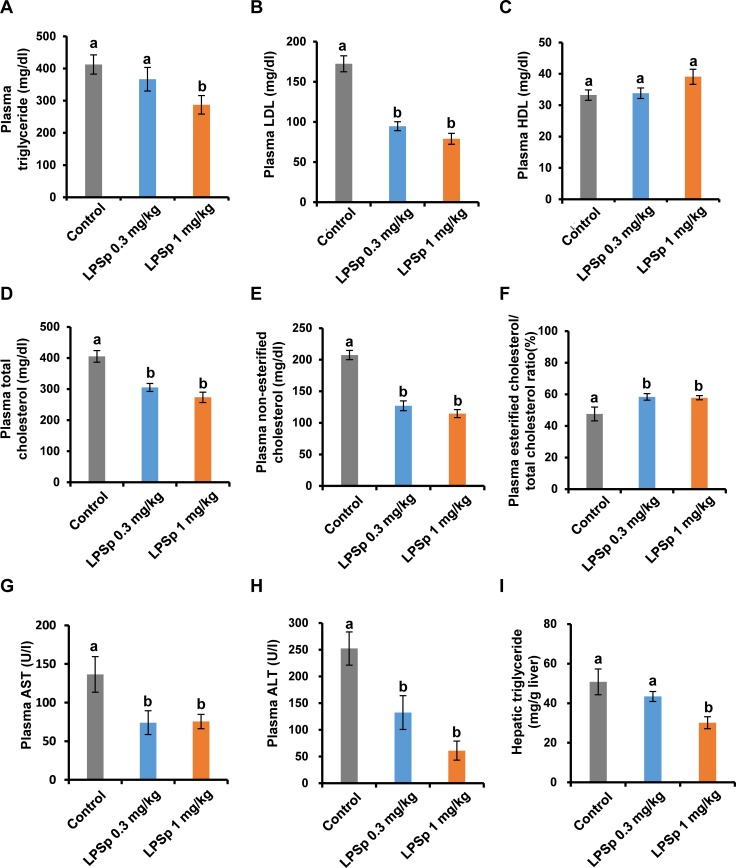
Effects of orally administered LPSp on plasma lipid profiles, AST activity, ALT activity and hepatic triglyceride accumulation. (A–H) After the experimental period, plasma triglyceride, LDL, HDL, total cholesterol, non-esterified cholesterol, AST and ALT levels were measured using commercial kits. Plasma esterified cholesterol was calculated by subtracting non-esterified cholesterol from total cholesterol and then the ratio of plasma esterified cholesterol/total cholesterol was calculated. (I) The weight of excised liver tissue was recorded and the hepatic lipids were extracted. The hepatic triglyceride level was measured using a commercial kit and indicated as mg of triglyceride per g of liver weight. Values are presented as the mean ± SEM, *n* = 7–8. Different letters indicate statistically significant difference between treatments (*p* < 0.05, one way ANOVA, post-hoc Tukey test).

As shown in Fig 4, the mice treated with LPSp (1 mg/kg BW/day) had significantly lower levels of plasma triglyceride (*p* < 0.05, vs. the control group) (Fig 4A), LDL (*p* < 0.01) (Fig 4B), total cholesterol (*p* < 0.01) (Fig 4D) and non-esterified cholesterol (*p* < 0.01) (Fig 4E) and higher levels of esterified/total cholesterol ratio (*p* < 0.05) (Fig 4F). The plasma HDL level tended to be greater in the LPSp-treated groups (by 18%; 39.1 ± 2.4 pg/ml) as compared to the control group (33.2 ± 1.6 pg/ml), but this difference was not statistically significant (*p* = 0.10) (Fig 4C). Liver plays an important role in the maintenance of normal lipid metabolism and glucose tolerance [29]. HFD-induced impairment of liver function in mice is characterized by elevated plasma AST and ALT activities and hepatic lipid accumulation [30]. As expected, there was a remarkable reduction in plasma AST (Fig 4G) and ALT (Fig 4H) and hepatic triglyceride accumulation (Fig 4I) (*p* < 0.01). In the lower dose LPSp group, significant differences were observed in LDL (*p* < 0.01, vs. the control group), total cholesterol (*p* < 0.01), non-esterified cholesterol (*p* < 0.01), esterified/total cholesterol ratio (*p* < 0.05), AST (*p* < 0.05) and ALT (*p* < 0.05). Taken together, these findings demonstrated that oral administration of LPSp prevented the HFD-induced weight gain and development of atherosclerotic plaque, and improved the glucose tolerance, plasma lipid profiles and liver functions. Therefore, we next investigated whether orally administered LPSp was effective in regulating HFD-induced inflammatory responses.

**Fig 5:**
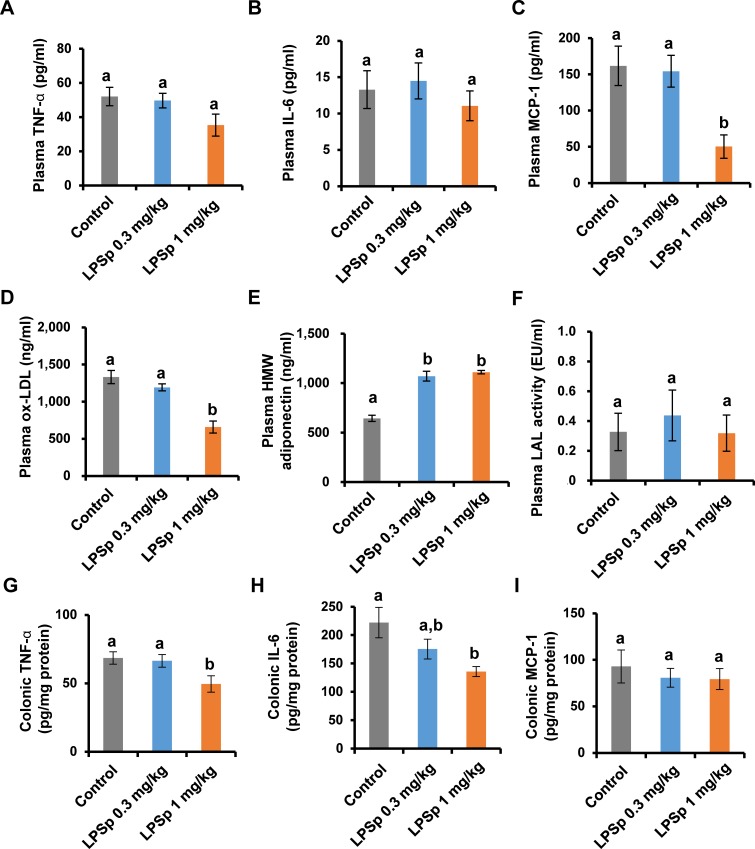
Effects of orally administered LPSp on inflammatory cytokines, chemokines, ox-LDL, HMW-adiponectin and LAL coagulation activity. (A–E) The plasma levels of TNF-α, IL-6, MCP-1, ox-LDL and HMW-adiponectin were determined using commercial ELISA kits, *n* = 7–8. (F) In order to evaluate the total abundance of LPS in plasma, the LAL coagulation activity, which is correlated with the LPS concentration, was measured, *n* = 3. (G–I) The colonic expression of TNF-α, IL-6 and MCP-1 were determined using commercial ELISA kits, *n* = 8. The concentration of cytokine is reported as picograms of cytokine relative to protein content. Values are presented as the mean ± SEM and different letters indicate statistically significant difference between treatments (*p* < 0.05, one way ANOVA, post-hoc Tukey test).

The expression of the plasma pro-inflammatory cytokine TNF-α tended to be lower (by 32%, 35.3 ± 6.4 pg/ml) in the LPSp (1 mg/kg BW/day)-treated group as compared to the control group (52.0 ± 5.4 pg/ml), but this difference was not significant (*p* = 0.11, Fig 5A). Also, there were no significant differences in plasma IL-6 levels (Fig 5B). Oral administration of LPSp (1 mg/kg BW/day) was effective (*p* < 0.01) in lowering the plasma levels of MCP-1, which is a biomarker of monocyte-related inflammation [31] (Fig 5C). Interestingly, oral administration of LPSp (1 mg/kg BW/day) profoundly decreased plasma ox-LDL levels (*p* < 0.01, vs. the control group) (Fig 5D). Moreover, the plasma level of HMW-adiponectin, which is inversely correlated with glucose tolerance and inflammation [32], was significantly elevated in both LPSp-treated groups as compared to the control group (*p* < 0.01) (Fig 5E). In order to evaluate the total amount of plasma LPS, the LAL coagulation assay was performed. The standard curve was determined using the standard LPS and showed a high correlation coefficient (r^2^ = 0.986) (S4 Fig and S1 Table). There were no significant differences between groups (Fig 5F). The analysis of colonic cytokine expression showed that the LPSp treatment (1mg/kg BW/day) was effective in lowering TNF-α and IL-6 expression (*p* < 0.05 vs. the control group) (Fig 5G and 5H). There were no significant changes in the colonic MCP-1 (Fig 5I).

### Measurement of superoxide anion production, myeloperoxidase activity and phagocytosis in the peripheral blood sample

**Fig 6:**
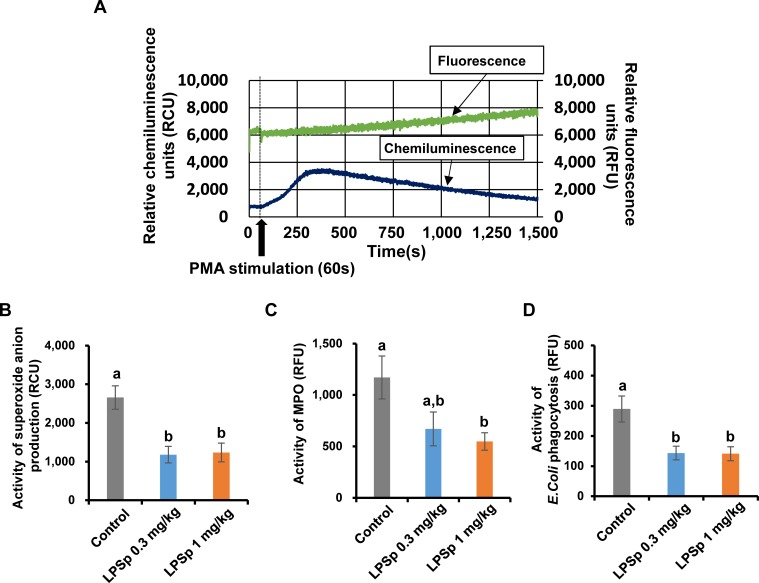
Effects of orally administered LPSp on superoxide anion production, MPO activity and phagocytosis in the peripheral blood sample. (A) Representative time change graphs of chemiluminescence and fluorescence in the leukocyte fraction obtained from the peripheral blood sample, following PMA stimulation. Superoxide anion production and MPO activity were simultaneously measured using a real-time monitoring system of chemiluminescence and fluorescence (CFL-P2200, Hamamatsu Photonics K.K.). The blue and green colored lines indicate the relative chemiluminescence units (RCU) and relative fluorescence units (RFU) curves, respectively. Data acquisition was performed every 0.5 s. (B) Superoxide anion production and (C) MPO activity after PMA stimulation was calculated by subtracting the basal intensity from the peak intensity, respectively. (D) The phagocytic activity against an *E*. *coli* bioparticles in the blood was calculated by subtracting the RFU of a 4°C negative control sample from that of a 37°C incubated sample. Values are presented as the mean ± SEM, *n* = 7–8. Different letters indicate statistically significant difference between treatments (*p* < 0.05, one way ANOVA, post-hoc Tukey test).

**Fig 7:**
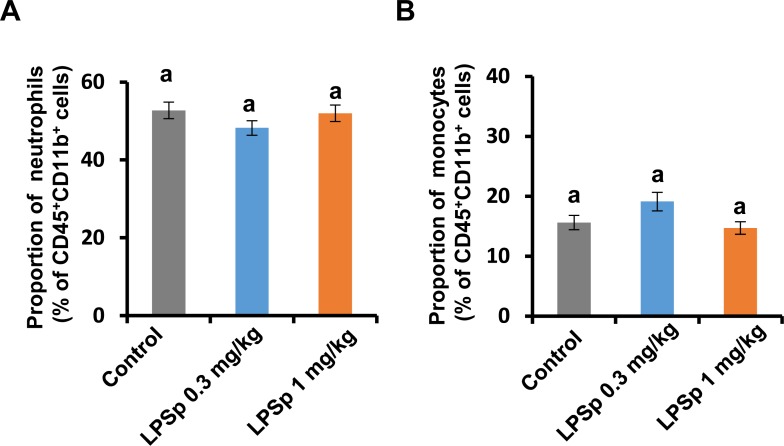
The population of neutrophils and monocyte in the peripheral blood sample. The population of (A) CD45^+^CD11b^+^Ly-6G^+^ neutrophils and (B) CD45^+^CD11b^+^Ly-6G^-^Ly-6C^+^ monocytes are indicated as the percentage of CD45^+^CD11b^+^ cells. Values are presented as the mean ± SEM, *n* = 8. No significant changes were observed among the groups (one way ANOVA, post-hoc Tukey test).

It is reported that upregulation of vascular inflammation is triggered by excessive oxidative activity of circulating white blood cells, induced by hypercholesterolemia or liver dysfunction in apoE-deficient mice [33]. Cellular oxidative responses in leukocytes are mainly regulated by oxidative bursts, including reactive oxygen species (ROS) production and MPO-mediated oxidative reactions [34]. To evaluate the oxidative burst activity in the leukocyte fraction obtained from the peripheral blood sample, a real-time dual monitoring device of chemiluminescence/fluorescence developed by Hamamatsu Photonics K.K. [23,24] was applied here. The device was developed to monitor the changes in chemiluminescence intensity by superoxide anion (O_2_^●-^) production and the changes in fluorescence intensity by MPO reaction after PMA stimulation at every 0.5 s. Representative results of the analysis are shown in Fig 6A. As shown in Fig 6B, the O_2_^●-^ production by PMA stimulation was lower in both LPSp-treated groups as compared to the control group (*p* < 0.01). Likewise, the PMA-induced MPO activity was lower in the LPSp (1 mg/kg BW/day)-treated group (*p* < 0.05) (Fig 6C). The low-dose LPSp (0.3 mg/kg BW/day) showed a tendency to decrease the MPO activity (*p* = 0.12). In addition, oxidative burst activity is well associated with phagocytic activity, which maintains immune homeostasis against xenobiotics [35]. Phagocytic activity was measured using *E*. *coli* bioparticles, which showed a significant reduction in both LPSp-treated groups as compared to the control group (*p* < 0.05) (Fig 6D). In addition, there were no significant differences among the groups in the proportion of neutrophils (defined as CD45^+^CD11b^+^Ly-6G^+^) or monocytes (CD45^+^CD11b^+^Ly-6G-Ly-6C^+^) (Fig 7).

### Analysis of gut microbiota profiles

**Fig 8:**
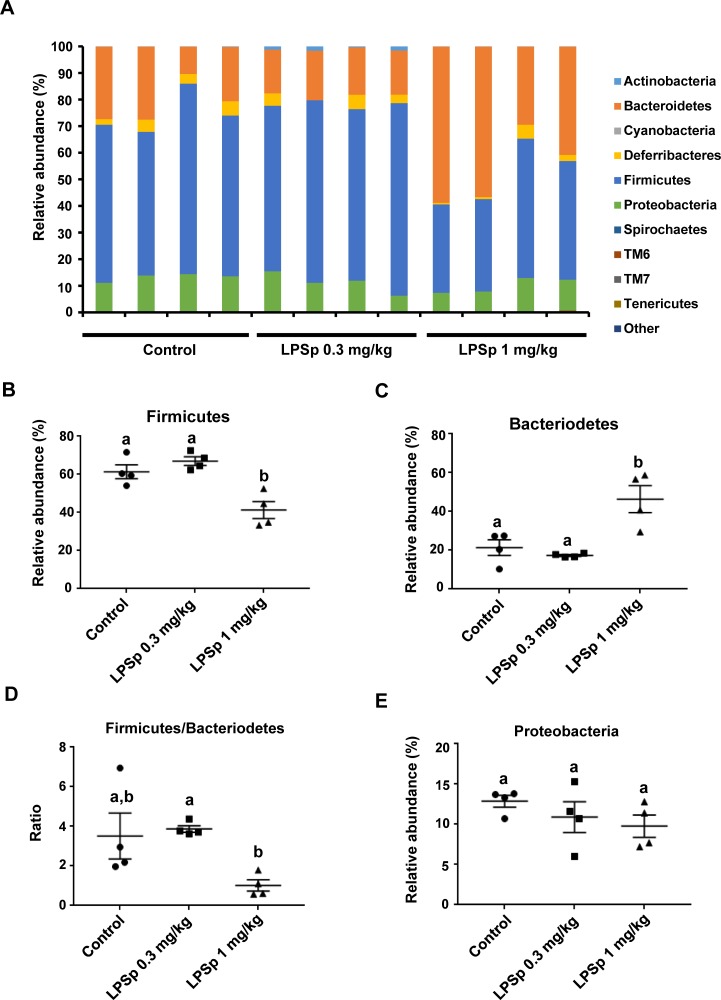
Effects of orally administered LPSp on gut microbiota composition. (A) Variation in bacterial community compositions in stool samples at the phylum levels. (B–E) The relative abundance (%) of Bacteroidetes, Firmicutes and Proteobacteria, and the ratio of Bacteroidetes to Firmicutes. Values are presented as the mean ± SEM, *n* = 4. Different letters indicate statistically significant difference between treatments (*p* < 0.05, one way ANOVA, post-hoc Tukey test).

The gut microbiota composition was analyzed by extracting microbial DNA from stool samples, which was then subjected to Miseq 16S rRNA gene sequencing. As shown in Fig 8A, the relative abundance of bacteria was calculated at the phylum level. As compared to the control group, oral administration of LPSp (1 mg/kg BW/day) led to a significant decrease in Firmicutes sp. (*p* < 0.01) (Fig 8B) and an increase in Bacteroidetes sp. (*p* < 0.05) (Fig 8C), and showed a trend toward a decrease in the abundance ratio of Firmicutes to Bacteriodetes (*p* = 0.08) (Fig 8D). Between the two LPSp-treated groups, higher dose of LPSp (1 mg/kg BW/day) was more effective in decreasing Firmicutes sp. (*p* < 0.01 vs. the LPS 0.3 mg/kg BW/day group), increasing Bacteroidetes sp. (*p* < 0.05) and decreasing the ratio of Firmicutes to Bacteriodetes (*p* < 0.05). There were no significant differences among the groups in the relative abundance of Proteobacteria (Fig 8E).

## Discussion

The results of the present study demonstrated that oral administration of LPSp for 16 weeks had effectively decreased the gain in BW, improved glucose tolerance and plasma lipid profiles, and reduced development of atherosclerotic plaque in HFD-fed apoE-deficient (BALB/c.KOR/StmSlc-*Apoe*^*shl*^) mice. This mouse strain [36] was developed as one congenic mouse model of spontaneously hyperlipidemia by Matsushima and co-workers. The mice are deficient in apoE expression, and show development of atherosclerotic lesion in the aorta with aging and similar pattern of serum lipoproteins to one common apoE knockout (Apoe^*tm1Unc*^) mice [36]. In addition, chronic HFD feeding promotes hyperlipidemia, oxidative stress and atherosclerosis progression in BALB/c.KOR/StmSlc-*Apoe*^*shl*^ mice [37]. The present results showed that the ratio of atherosclerotic plaque lesion in the mice (*n* = 24) had a significant (*p* < 0.05) positive correlation with major metabolic parameters including liver weight (Pearson's correlation coefficient (r) = 0.43), fasting insulin (r = 0.46), HbA1c (r = 0.43), plasma triglyceride (r = 0.51), total cholesterol (r = 0.56) and plasma LDL (r = 0.53), and had a trend toward a positive correlation with BW (r = 0.40, *p* = 0.06), white adipose tissue weight (r = 0.35, *p* = 0.09) (S2 Table), suggesting the development of atherosclerosis can be considered to be associated with impaired glucose tolerance and lipid metabolisms in the HFD-fed BALB/c.KOR/StmSlc-*Apoe*^*shl*^ mice.

Further, the analysis of inflammatory mediators showed that LPSp treatment significantly reduced plasma MCP-1 expression. In addition, the results of our LAL assay showed no significant difference in plasma LAL activity between groups, suggesting that microbiome-derived LPS translocation into the bloodstream might not be enhanced by LPSp treatment. However, it has been reported that the reactivity of LPS to LAL reagents depends on the genus and species of the source gram-negative bacteria [38]. The present LAL test seems to be limited to evaluating the total abundance of plasma LPS, because the LAL assay was not able to distinguish between LPSp and endogenous LPS. In addition, LPS binding plasma components (lipoproteins and platelets) also alter the interactions of LPS with LAL reagents to cause high measurement variations [39]. Hence, further studies are needed to develop a reliable assay for LPSp-specific quantification to follow the fate of orally administered LPSp.

To elucidate the possible mechanisms underlying the anti-atherosclerotic effect of LPSp through a reduction of the HFD-induced inflammatory responses, the effects of LPSp on oxidative burst activity were investigated in the leukocytes obtained from the peripheral blood. During an oxidative burst in neutrophils and monocytes, the two major enzymes NADPH oxidase and MPO are involved in oxidative reactions to produce ROS [34]. These oxidants are involved in ox-LDL generation and the excess ox-LDL induces an increase in oxidative burst activity as in a negative feedback loop [34]. In the present study, the oxidative burst activity in the leukocyte sample was assessed by measuring two key activities (O_2_^●-^ production and MPO responses). The PMA-stimulated O_2_^●-^ production and MPO activity were significantly reduced in mice following oral administration of LPSp without affecting the numbers of neutrophils and monocytes in the peripheral blood. The activity of oxidative burst-related phagocytosis was also decreased. These findings suggested that the lowering effect of plasma ox-LDL levels by orally administered LPSp may be mediated partly *via* the reduction of oxidative burst responses. Oral administration of β-glucan, which is considered as an immunostimulant and non-absorbable polysaccharide, was shown to decrease HFD-fed induced oxidative stress and development of atherosclerotic plaques in a rodent model [40]. These beneficial effects may be results of improvement of microbiota profiles and intestinal immune system [41]. Similarly, a plausible underlying mechanism of orally administered LPSp in the reduction of HFD-induced oxidative stress is considered to be an indirect effect on the gastrointestinal tract.

The changes to intestinal microbiota profiles and intestinal mucosa immunity are associated with inflammatory diseases, such as diabetes and atherosclerosis. Several studies using apoE-deficient mice reported that the abundance of bacteria belonging to the phylum Firmicutes is increased, that of the phylum Bacteriodetes is decreased or the ratio of Firmicutes to Bacteriodetes is increased with the development of atherosclerosis, and anti-atherosclerotic supplements can improve the microbiota profiles [42,43]. Consistent with these reports, the present data showed an improvement in the abundance of Firmicutes and Bacteroidetes sp. and a trend in a decrease of the Firmicutes/Bacteroidetes ratio by LPSp. In addition, the expression of colonic pro-inflammatory cytokines (TNF-α and IL-6) was significantly reduced, suggesting orally administered LPSp might involve in regulation of microbiota profiles and intestinal inflammation. Other study reported that the transplantation of Bacteroidetes rich mice microbiota ameliorated HFD-induced glucose intolerance in mice, but its underlying molecular mechanisms are still unknown [44]. Recent studies have shown that a heat shock protein (acts as TLR 4 agonist) can activate T regulatory (Treg) cells in intestine and blood, resulting in a decrease in atherosclerotic plaque size when administrated orally to mice [45]; LPS-stimulated Treg cells can interact with blood neutrophils to induce suppression of neutrophil-mediated inflammatory responses in *in vitro* [46]. However, it is still unclear that the mechanisms underlying an anti-atherosclerotic effect by activated Treg cells via regulating intestinal mucosa immunity including microbiota compositions and inflammatory responses. During progression of atherosclerosis, apoE-deficient mice shows obviously decreased number in Treg cells as compared with wild type littermates [47]. HFD treatment is also contributes to alter Treg expression in the wild type mice [48]. Therefore, further studies are required to elucidate whether orally administered LPSp can regulate Treg cells expression and/or microbiota profiles to prevent atherosclerosis development in an animal model of atherosclerosis without HFD treatment or with germ-free condition.

It must be pointed out that LPS injection is known to aggravate atherosclerosis. However, in *in vivo* studies, the route of LPS injection is limited to intravenous or intraperitoneal, and the doses of LPS usually range between 0.5 mg/kg and 2.5 mg/kg BW, which is sufficient to cause atherosclerosis in apoE-deficient mice [49–51]. On the other hand, oral administration of LPSp at a high dose (300 mg/kg BW, 4 weeks) caused no significant hepatotoxicity or nephrotoxicity in rat [52]. Orally administered *E*.*coli* LPS was found to regulate a mucosal immunity in the intestine and enhance innate immune defense without activating serum TNF-α expression in mice [53]. Fukasaka *et al*. demonstrated that sublingual administration of LPSp promoted TLR4-dependent priming of dendritic cells, which resulted in the activation of host immune responses against pathogen infection in mice [12]. These reports suggest that physiological effects after oral or sublingual LPS treatment should be considered to be regulated by different mechanisms as compared with intravenous or intraperitoneal injection.

In summary, the results of the present study provide new evidence that oral administration of LPSp significantly inhibited the development of atherosclerotic plaque via improving glucose tolerance, plasma lipid profiles, decreasing pro-inflammatory mediator production, and reduced oxidative burst activities in the peripheral blood. It has been reported that LPS was detected in various Chinese herbal medicines and dietary cereals (1 to 100 μg/g of dry weight), and was considered to be derived from Gram-negative bacteria that are symbiotic to plants [54]. A herbal medicine, Juzen-taiho-to, has been shown to prevent hepatic inflammation when administered orally to a mouse model of NASH [55] and its active compound was determined as LPS mainly derived from genus *Rahnella* [56]. In the future, we would like to evaluate beneficial effect of agricultural foods that contain symbiotic bacteria-derived LPS on atherosclerosis.

## Supporting information

S1 Fig The stability of LPSp in drinking water. Values are presented as the mean ± SD, *n* = 3. No significance difference was observed between groups (two way ANOVA, post-hoc Tukey test).
(PDF)

S2 Fig The gated strategy of neutrophils and monocytes. (A-D) The gated strategy of neutrophils (CD45^+^CD11b^+^Ly-6G^+^ cells) and monocytes (CD45^+^CD11b^+^Ly-6G^-^Ly-6C^+^ cells). (E) Re-analysis of monocytes (blue dots) and neutrophils (green dots) in FS-SS plot to check their distribution. FS: forward scatter, SS: side scatter.
(PDF)

S3 Fig Weekly food intake during the experiment. Values are presented as the mean ± SEM, *n* = 8. No significance difference was observed between groups (two way ANOVA, post-hoc Tukey test).
(PDF)

S4 Fig The standard curve of the present LAL assay. The standard curve was plotted with log concentration of each standard LPS against log reaction time (*n* = 3). R^2^ indicates the coefficient of determination.
(PDF)

S1 Table The standard data of the present LAL assay. (DOCX)

S2 Table Pearson's correlation coefficients among the ratio of atherosclerotic plaque lesion and major metabolic parameters. (DOCX)
