# Supplementary figures and images for: Oral administration of Pantoea agglomerans-derived lipopolysaccharide prevents development of atherosclerosis in high-fat diet-fed apoE-deficient mice via ameliorating hyperlipidemia, pro-inflammatory mediators and oxidative responses
Source: PLoS One. 2018 Mar 27;13(3):e0195008. doi: 10.1371/journal.pone.0195008 (PMC5871011; doi:10.1371/journal.pone.0195008)

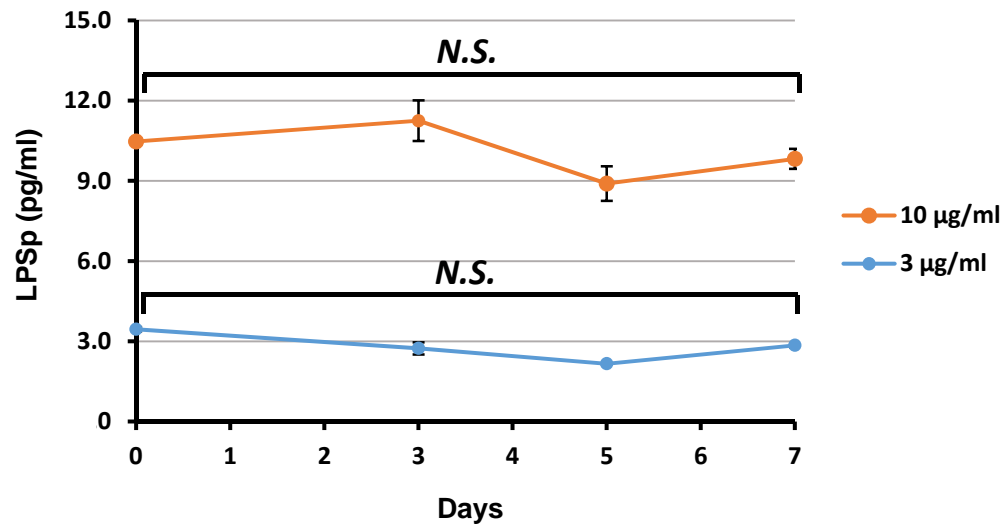

Supplement: S1 Fig — Values are presented as the mean ± SD, n = 3. No significance difference was observed between groups (two way ANOVA, post-hoc Tukey test). (PDF) [file pone.0195008.s001.pdf]

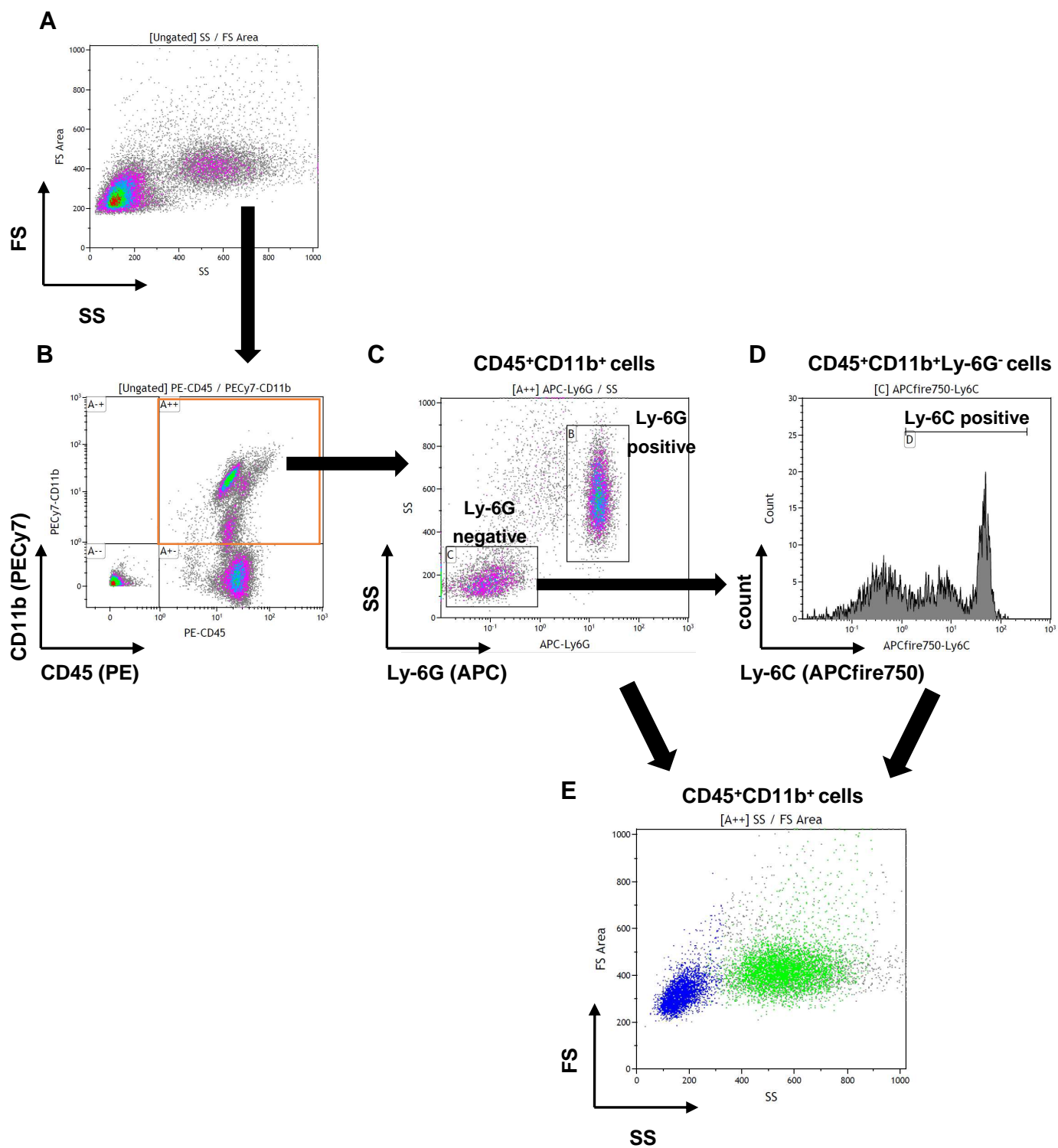

Supplement: S2 Fig — (A-D) The gated strategy of neutrophils (CD45+CD11b+Ly-6G+ cells) and monocytes (CD45+CD11b+Ly-6G-Ly-6C+ cells). (E) Re-analysis of monocytes (blue dots) and neutrophils (green dots) in FS-SS plot to check their distribution. FS: forward scatter, SS: side scatter. (PDF) [file pone.0195008.s002.pdf]

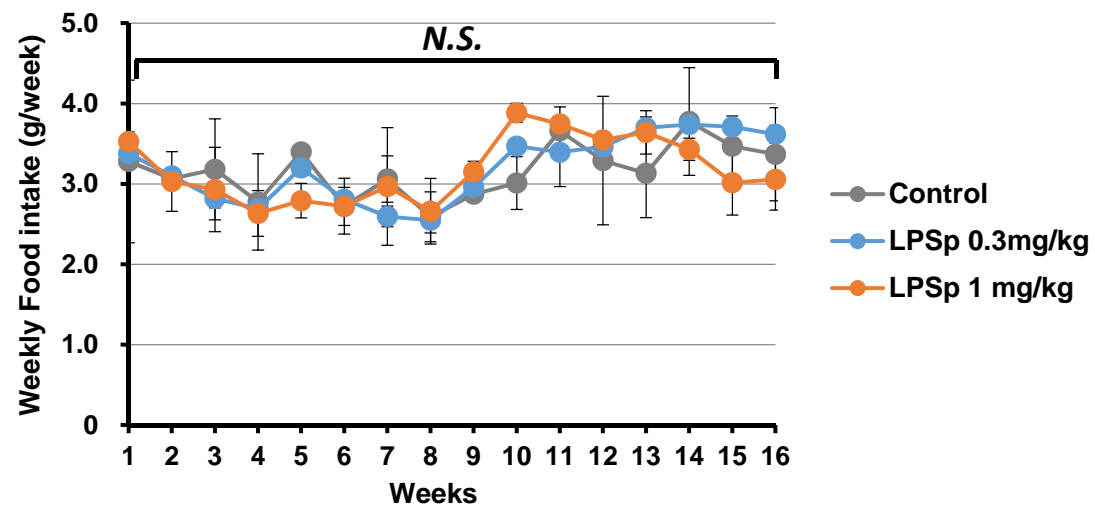

Supplement: S3 Fig — Values are presented as the mean ± SEM, n = 8. No significance difference was observed between groups (two way ANOVA, post-hoc Tukey test). (PDF) [file pone.0195008.s003.pdf]

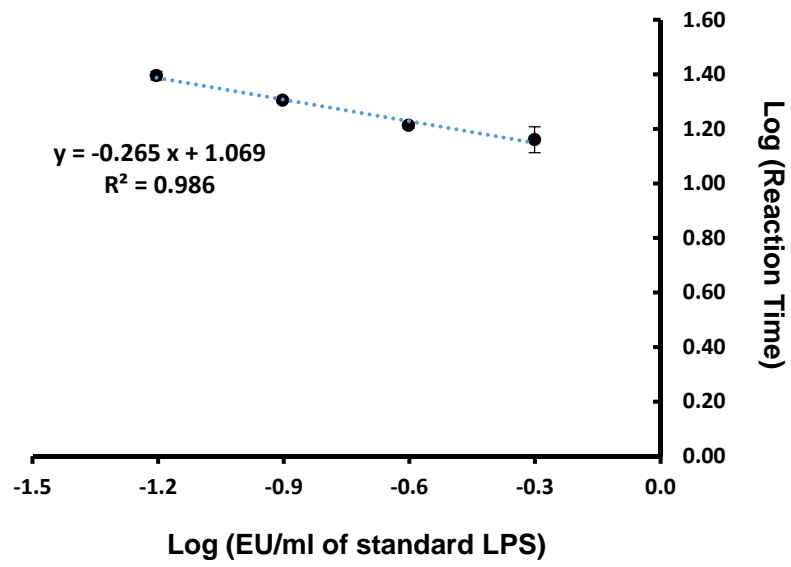

Supplement: S4 Fig — The standard curve was plotted with log concentration of each standard LPS against log reaction time (n = 3). R2 indicates the coefficient of determination. (PDF) [file pone.0195008.s004.pdf]
