# Supplementary material for: Oral administration of Pantoea agglomerans-derived lipopolysaccharide prevents development of atherosclerosis in high-fat diet-fed apoE-deficient mice via ameliorating hyperlipidemia, pro-inflammatory mediators and oxidative responses
Source: PLoS One. 2018 Mar 27;13(3):e0195008. doi: 10.1371/journal.pone.0195008 (PMC5871011; doi:10.1371/journal.pone.0195008)
Supplement: S1 Table — (DOCX) [file pone.0195008.s005.docx]

| EU/ml | Log (EU/ml) | Reaction time  (*n* = 3) | | | Log (reaction time)  (*n* = 3) | | | Average | SD |
| --- | --- | --- | --- | --- | --- | --- | --- | --- | --- |
| 0.500 | -0.301 | 15.8 | 12.8 | 15.0 | 1.20 | 1.11 | 1.18 | 1.16 | 0.05 |
| 0.250 | -0.602 | 16.0 | 16.8 | 16.2 | 1.20 | 1.23 | 1.21 | 1.21 | 0.01 |
| 0.125 | -0.903 | 19.8 | 20.4 | 20.4 | 1.30 | 1.31 | 1.31 | 1.31 | 0.01 |
| 0.0625 | -1.204 | 24.0 | 25.8 | 24.8 | 1.38 | 1.41 | 1.39 | 1.40 | 0.02 |
