# Supplementary material for: Oral administration of Pantoea agglomerans-derived lipopolysaccharide prevents development of atherosclerosis in high-fat diet-fed apoE-deficient mice via ameliorating hyperlipidemia, pro-inflammatory mediators and oxidative responses
Source: PLoS One. 2018 Mar 27;13(3):e0195008. doi: 10.1371/journal.pone.0195008 (PMC5871011; doi:10.1371/journal.pone.0195008)
Supplement: S2 Table — (DOCX) [file pone.0195008.s006.docx]

| Metabolic parameters | Pearson's correlation coefficient | *p* value |
| --- | --- | --- |
| Body weight | 0.396 | 0.056 |
| Liver weight | 0.425 | 0.038 |
| Adipose tissue weight | 0.351 | 0.092 |
| Fasting blood glucose | 0.244 | 0.251 |
| Plasma insulin | 0.464 | 0.022 |
| HbA1c | 0.427 | 0.038 |
| Plasma triglyceride | 0.508 | 0.011 |
| Plasma LDL | 0.530 | 0.008 |
| Plasma HDL | -0.126 | 0.557 |
| Plasma total cholesterol | 0.564 | 0.004 |
